# A Study on the Effect of Macro- and Micro- Nutrients on *Nannochloropsis oceanica* Growth, Fatty Acid Composition and Magnetic Harvesting Efficiency

**DOI:** 10.3390/plants9050660

**Published:** 2020-05-23

**Authors:** Maria G. Savvidou, Elenitsa Boli, Dimitrios Logothetis, Theopisti Lymperopoulou, Angelo Ferraro, Vasiliki Louli, Diomi Mamma, Dimitris Kekos, Kostis Magoulas, Fragiskos N. Kolisis

**Affiliations:** 1 Biotechnology Laboratory, School of Chemical Engineering, National Technical University of Athens, 9 Iroon Polytechniou Str, Zografou Campus, 15780 Athens, Greece; msavvid@central.ntua.gr (M.G.S.); ferraro@eie.gr (A.F.); dmamma@chemeng.ntua.gr (D.M.); kekos@chemeng.ntua.gr (D.K.); 2 Laboratory of Thermodynamics and Transport Phenomena, School of Chemical Engineering, National Technical University of Athens, 9 Iroon Polytechniou Str, Zografou Campus, 15780 Athens, Greece; bolieleni@chemeng.ntua.gr (E.B.); dimitrioslogo@yahoo.com (D.L.); svlouli@chemeng.ntua.gr (V.L.); mag@chemeng.ntua.gr (K.M.); 3 Environment and Quality of Life Center, School of Chemical Engineering, National Technical University of Athens, 9 Iroon Polytechniou Str, Zografou Campus, 15780 Athens, Greece; veralyb@chemeng.ntua.gr

**Keywords:** starvation, fatty acids profile, microalgae, magnetic nanoparticles, culture medium

## Abstract

The effect of iron, manganese, phosphorus and nitrogen on growth and lipid synthesis of the microalgae *Nannochloropsis oceanica* CCMP1779, as well as their impact on the magnetic harvesting efficiency, are examined under their depriving cell culture conditions. Herein, it is demonstrated that nitrogen and manganese depletion primarily reduced cell growth while phosphorus and iron restriction led to higher dry biomass. Subsequently, the role of those nutrients on fatty acids profile was examined. Phosphorus and nitrogen restriction resulted in lower and higher lipid content, respectively. High amounts of polyunsaturated fatty acids like eicosapentaenoic acid are produced under iron and manganese depletion. Phosphorus deprivation favors monounsaturated fatty acids such as C18:1 and C16:1, while nitrogen restriction favors saturated fatty acid production like C14:0, C16:0 and C18:0. Since the presence/absence of macro- and micro-elements may affect the overall electrostatic charges on the outmost microalgae surface, it was also analyzed how these elements affect the magnetic harvesting efficiency. Results showed that phosphorus deprivation led to the best magnetic harvesting efficiency of *N. oceanica* cells (93%) as compared to other nutrient starvation as well as standard medium.

## 1. Introduction

Demand for algae-based lipids is increasing and can be satisfied by an efficient lipid biosynthesis using proper nutrients as well as by optimizing harvesting strategies that lead to high cell/biomass recovery. Different physico-chemical conditions such as temperature, stress, light intensity, culture time, organic carbon and inorganic nutrients including iron (Fe), phosphorous (P), nitrogen (N), manganese (Mn), zinc (Zn), sulfur (S), cobalt (Co) and others, affect and regulate growth and lipid accumulation of several microalgae species [1].

The significance of the above mentioned elements for microalgae growth is linked to their involvement in basal biological processes. It is known that Mn^+2^ is the catalyst of photosynthesis and the activator of microalgae enzymes like superoxide dismoutase [2,3]. Furthermore, the oxygen-evolving complex (OEC), which is involved in the photo-oxidation of water during the light reactions of photosynthesis, consists of a cluster of four manganese ions in its catalytic unit [4]. Iron has been demonstrated to be responsible for enzymatic activity, transport systems, nitrogen consumption and redox reactions [5]. Nucleic acid synthesis and chlorophyll production are regulated by nitrogen [6], while phosphorus is a key element for metabolism, energy transfer and nucleic acid composition of the algae cells [1].

Microalgae are a diverse group of microorganisms producing fatty acids, carotenoids and other bioactive compounds [7]. Correlation between presence of micro or macro nutrients and/or their concentration with growth rate and lipid production has been established in different microalgae species [1]. Specifically, higher Fe^+3^ concentrations increase the lipid content [8] while controversial results about the above increment in the genus *Scenedesmus* under Fe^+3^ deficiency have been stated [1]. The effect of iron on growth rates is also under examination [9]. Regarding heavy metals, higher concentration of Mn^+2^ and Co^+2^ in the culture medium enhanced the lipid productivity of *Chlorella vulgaris* [10]. In many microalgal strains, including *N. oceanica*, nitrogen starvation results in cessation of cell divisions and induction of quiescence, leading to a significant reduction of growth rates [11]. Furthermore, nitrogen deprivation is related to enhanced lipid productivity in various algae strains [12]. On the other hand, phosphorus starvation may limit the growth but it triggers lipid production in *Chlorococcum infusionum* [13]. Combinatorial studies by simultaneously analyzing the presence or absence of specific elements like phosphorus and nitrogen signify the role of those nutrients towards lipid production [1].

Recently, magnetic nanoparticles have been used for algal biomass harvesting as an alternative to other well-known procedures such as centrifugation, filtration, flocculation, sedimentation, and flotation [14]. The magnetic harvesting strategy is simple and depends on algae cells surface interactions with magnetic nanoparticles that result in the aggregation and formation of a complex (cell–nanoparticles) with magnetic properties. A magnetic field attracts the aggregates leading to algae separation from the growth culture medium [15]. Surface functionalization of iron oxide (Fe_3_O_4_) particles [16] is extensively used in order to optimize the ability of particles to be adsorbed on the cell surface [15]. Magnetic nanoparticles are used in various biomedical applications and in bioseparation of pathogenic cells, viruses and proteins [16]. They present many advantages over the traditional methods like high efficiency, lower cost and less energy consumption while the whole procedure is faster [17]. Repulsion of the nanoparticles from the cells through surface charge or surface wetting properties leads to the dissociation of aggregates and the isolation of algae cells [16]. Proper solutions in suitable pH or even special formed nanocomposite like ZnO-Fe_3_O_4_ are used for better particles detachment from cell-surface and reprocess particles [14,16]. Several studies concerning microalgae harvesting with magnetic nanoparticles have been reported. Furthermore, the effects of various factors like temperature, pH, nanoparticles dosage, etc., have been extensively studied [18,19]. Nevertheless, to the best of our knowledge there are no studies focused on the improvement of magnetic harvesting by altering the medium compositions.

In this study, in order to optimize the best harvesting condition while preserving biomass quality, the dry biomass yield, the growth rate, the lipid content and the amount of chlorophyll a were analyzed in *Nannochloropsis oceanica* CCMP1779 cultured in specific nutrient deprivation. *Nannochloropsis* is a well-known microalgae species that has been used to produce fish feed, biofuels and pharmaceutical compounds [20,21]. The depletion effect of single trace micronutrients like Fe^+3^ and Mn^+2^ as well as single nutrients like P and N is investigated. In this work, P depletion resulted in high cell separation, less stirring time and simultaneously enhanced the metabolism of *N. oceanica* by favoring MUFAs lipid production.

## 2. Results and Discussion

### 2.1. Effect of Macro- and Micro-Elements on N. oceanica Growth, Lipid Biosynthesis and Chlorophyll Content

**Figure 1:**
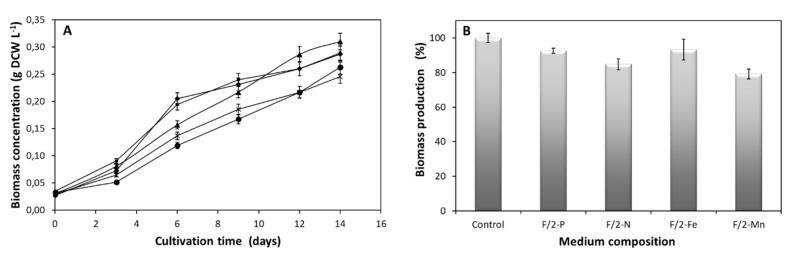
(**A**) Effect of modified medium lacking each of the following components (◆) NaH_2_PO_4_ (F/2-P), (●) NaNO_3_ (F/2-N), (■) FeCl_3_ (F/2-Fe), (**X**) MnCl_2_ (F/2-Mn), (▲) F/2 medium (control) on *N. oceanica* biomass during 14 days of cultivation. (**B**) Biomass production of *N. oceanica* CCMP1779 in F/2 medium (control) vs. modified medium lacking each of the following components: NaH_2_PO_4_ (F/2-P), NaNO_3_ (F/2-N), FeCl_3_ (F/2-Fe), MnCl_2_ (F/2-Mn). The biomass production was estimated as a percentage of each one towards the growth in complete culture medium (X_max_, control = 0.31 g L^−1^). Culture conditions: 24 h light, 100 μmol m^−2^ s^−1^ light intensity, shaking 130 rpm and temperature 20 ± 1°C. Data correspond to the mean ± SD of three independent experiments.

Aiming to identify the effects of specific nutrients on *N. oceanica* basal biological processes, different formulations of culture medium were used. Cell growth, lipid and chlorophyll content were calculated on day 14 of the cultivation and compared to the respective levels from cells grown on complete medium. At day 14, cells were at the exponential phase of growth as showed in Figure 1A, thus all metabolic activities were measured at this cultivation point. Figure 1B describes the percentage biomass concentration at day 14. In each experimental point one essential medium component such as NaH_2_PO_4_, NaNO_3_, FeCl_3_ and MnCl_2_ was absent. Single N- or Mn-depletion led to reduced growth by 15.2% and 20.8%, respectively (Figure 1B), while restriction of P or Fe resulted in a minor reduction of 7.5% and 6.8%, respectively (Figure 1B) measuring the biomass production. The findings of this study imply that N and Mn are essential for *N. oceanica* growth, whereas depletion of P or Fe can be better tolerated. This is likely due to the fact that N and Mn are related to cell cycle progression. As a consequence of the growth, Fe and P depletion demonstrate higher dry biomass (X_max =_ 0.29 g L^−1^), compared to the N or Mn deprivation (Table 1), with P absence adding a slight advantage (P_max =_ 18.55). Alvarez-Díaz and colleagues, demonstrated that the effect of P depletion can be compensated by the presence of organic N [22]. This fact supports the maximum dry biomass observed by *N. oceanica* under P restriction in our studies.

Chen et al. refer that Fe deprivation reduced growth of *Dunaliella tertiolecta* almost five times more than in our case [9], while lower P concentrations limit the growth of *Chlorella vulgaris* [1]. Nitrogen deprivation significantly reduced cell growth of fresh water microalgae and *Chlorella pyrenoidosa* [1]. Expansion of the cell phases and even arrest to G1 phase happens upon nitrogen depletion [23]. Manganese depletion led to similar phenotypes of arrested growth [24]. The above evidence can explain the reduction of growth under N or Mn depletion in *N. oceanica* based on the perturbation of cell cycle progression.

Concerning Mn, studies in *Chlorella vulgaris* reveal analogous effect as in *N. oceanica* since Mn depletion resulted in 20% growth reduction [10]. Manganese effect on *N. oceanica* cell growth can be explained also by its requirement as a constituent of metalloenzymes, proteins and vitamins which regulate algal metabolism [10]. The findings of the current study are in agreement with well-known effects of N and Mn deprivation on microalgae growth and further confirm that the growth of *N. oceanica* microalgae species, and probably the entire microalgae family, are negatively affected by these two elements.

Specific growth rates followed the trend of biomass as showed in Table 1. Analyzing the biomass production parameters for each one of the withdrawn elements, it is verified that Fe depletion resulted in higher growth rate (μ = 0.286, *p*-value < 0.01) (Table 1), while P restriction did not affect this factor (μ = 0.274, *p*-value > 0.01). Furthermore, deprivation of N or Mn led to reduced growth rates (μ = 0.215, *p*-value < 0.01 and μ = 0.253, *p*-value < 0.01, respectively). The above outcomes are in good accordance with previous literature data that highlight the small reduction of biomass in the case of Fe and P depletion and at the same time the higher growth reduction under Mn or N restriction.

In general, Fe is necessary for increasing growth of microalgal cells. Studies of *Dunaliella tertiolecta* [9] demonstrate the necessity of Fe for a normal growth rate. The increased growth rate under Fe deprivation in our study can be explained by the use of the intracellular stored Fe levels since this micronutrient is critical for the cell metabolism and is usually stored as ferritin [25]. The unchanged growth rate in the case of P restriction can be explained by the cell ability to store P as metaphosphates or polyphosphates, and those stocks can be utilized upon P deprivation [1]. The use of intracellular reservoir of P may provide to *N. oceanica* the ability to maintain its growth rates and have just a small reduction of growth, at least for a limited time-frame.

The four elements analyzed in this study can regulate both quality and quantity of lipid production in a different manner, since under phosphorus-depleted conditions, the total lipid content was reduced (Y = 23.7%) (Table 1) compared to other nutrient restrictions, despite the almost unchanged growth rate, while under nitrogen-depleted conditions the highest lipid content was achieved (Y = 41.5%) (Table 1) to the detriment of growth rate and biomass production. On the other hand, Fe and Mn restriction led to slightly decreased lipid content, with opposite effects on growth rate (Fe and Mn enhanced and reduced growth, respectively). The above findings display different metabolic responses (lipid production and growth) that may be regulated by single nutrients. Specifically, reduced lipid content upon Fe deprivation can be possibly explained by the effect of iron to some key genes like rbcL, accD, PAP1 targeting lipid synthesis [26]. Phosphorus is directly related to the lipid production due to its implication in fatty acid biosynthesis by activating genes like w-3 FAD [27]. Additionally, P is one of the most important elements of Krebs cycle so the reduced lipid yield in our experiments under P-depleted conditions can be explained by the reduced efficiency of Krebs cycle. Similar studies under the same depriving conditions in *Nannochloris atomus* and *Tetraselmis sp*. [28] demonstrated the same reduction, almost 40%, of lipid content.

Wang et al. demonstrated the clear effect of N deprivation on the increased lipid bodies in green alga *Chlamydomonas reinhardtii*, with 15 times more yield as compared to the presence of nitrogen [29]. *Chlorella vulgaris* exhibited an analogous and even more enhanced phenotype with respect to *N. oceanica* under N restriction [30]. A mechanistic analysis explaining the enhanced lipid content under nitrogen deprived conditions is based on the reduced amount of ACCase and hence its enzymatic activity, which catalyzes the conversion of acetyl-CoA to malonyl-CoA followed by fatty acid production, impairing in this way cell division and leading to enhanced lipid production [31]. Battah and coworkers, reported that *Chlorella vulgaris* increased lipid content by increasing Mn concentrations [10].

Chlorophyll production was more negatively influenced by P and by N depletion (Table 1) while Fe deprivation produced a different phenotype (Table 1). On the other hand, the depletion of Mn did not affect chlorophyll content. Juneja et al. state that the limitation of P diminishes the chlorophyll levels in *Chlamydomonas reinhartdtii* [32]. A reduced chlorophyll content under N-depleted conditions, compared to *N. oceanica*, has been observed in *Chlorella vulgaris* by Lv et al. [31]. These findings can be explained since N is essential for chlorophyll structure. Studies on the role of increasing Mn concentrations on photochemical efficiency in Arabidopsis demonstrate an inhibition of photosystem II through a down-regulation of photosystem I electron flow [33]. The experimental data presented in this study also revealed that the chlorophyll content observed at *N. oceanica*, under Mn-depleted conditions and under complete nutrient culture medium, was almost at the same levels as those reported in Millaleo et al. study [33]. The increased chlorophyll levels related to Fe depletion in *N. oceanica* are not in line with the role of Fe on photosynthesis, since this nutrient is responsible for the action of enzymes as coproporphyrinogen oxidase and aconitase [34]. In this study under Fe depletion the levels of chlorophyll are higher, possibly assessing supplementary metabolic pathways which are strongly activated and support chlorophyll production. In *Dunaliella salina* the presence of a thylakoid protein of 45 kDa, named Tidi, was identified as a chlorophyll a/b-binding protein supporting the maintenance of photosynthetic activity even under extreme iron deprivation [35]. Analogous proteins may support the photosynthetic system of *N. oceanica* upon the same depriving conditions.

### 2.2. Effect of Nutrients on Fatty Acid Profile

In most microalgae, lipid accumulation usually occurs as a response to stressful cultivation conditions or environmental stress. GC-MS analysis of the lipid content of *N. oceanica* enlightens the depletion effect of specific macro- and micro-nutrients on the final lipid profile compared to the cells grown in complete medium. In general, total fatty acids content is increased under P-, Fe- and Mn-depleted conditions (10.9%, 11.7% and 11.7%, respectively) (Table 2) despite the reduced lipid content, while a different phenotype revealed under N-depletion is in disagreement with increased lipid content. Total saturated fatty acids were reduced under P, Fe and Mn depletion (53.35%, 53.56% and 54.72%, respectively), while N restriction resulted in increased production (60.60%) (Table 2) since C14:0, C16:0 and C18:0 fatty acids were enhanced leading to the total increment of the saturated fatty acids (Table 2). Nitrogen deprivation is a crucial condition for biodiesel production since its quality is directly related to saturated and monounsaturated fatty acids [36], indeed saturated fatty acids have less effect in transesterification [37]. Similar fatty acid profile under N deprivation is reported by Yodsuwan et al. [37], which showed increased C16:0 and C14:0 levels. El-Kassas determined increased saturated fatty acids while monounsaturated were decreased in *Pichoclorum sp*. [36] and also Sun et al. verified that C16:0 and C18:0 increased in *Neochlorosis oleoabundans* HK-129 in a similar manner as in *N. oceanica* [26] under nitrogen deprived conditions.

Regarding P deprivation all saturated fatty acids were reduced with the exemption of C18:0 which was increased (Table 2). Furthermore, under Fe-depleted conditions, C12:0, C14:0 and C15:0 did not change, while C18:0 increased and C16:0 reduced. Analogous phenotypes happened under Mn depletion since C12:0 and C15:0 maintained their levels, while C14:0 and C18:0 increased, and C16:0 reduced leading to a total reduction of the saturated fatty acids. Manganese deprivation resulted in C14:0 and C18:0 increment for *C. vulgaris* [10] as in our study. As for monounsaturated fatty acids, P restriction revealed a different phenotype than the other three nutrients leading to a higher content of C16:1 (26.31%) and C18:1 (13.93%) (Table 2). As a consequence, based on our results and literature, any need for biodiesel rich in monounsaturated fatty acids can be fulfilled by P deprivation in *N. oceanica*. Higher levels of C16:1 under P deprivation have also been found in various *Nannochloropsis* species, as well as for C18:1 in *N. oculata* [38] under the same deprivation conditions.

Additionally, under P- or N-depleted conditions, the polyunsaturated fatty acid content was reduced by 6.42% and 7.11%, respectively, while Fe and Mn restriction increased significantly those fatty acid levels (14.76% and 16.98%, respectively) (Table 2). Specifically, C18:2, C20:4, C20:5 fatty acids were reduced under P or N deprivation, but were enhanced upon Fe or Mn depletion, leading to an overall increment of total polyunsaturated fatty acids. Concerning N starvation, the reduction of fatty acids could be due to the enhancement of triacylglycerol (TAG) biosynthesis which in microalgae is much more active under unfavorable environmental or stress conditions, thus leading to a reduction of free fatty acids [11]. Under Fe-depleted conditions, *N. oceanica* reflects a different phenotype than other microalgae such as *C. reinhardtii*, which showed increased C16:0 levels and in general more saturated than unsaturated lipids [39]. A similar phenotype regarding saturated and polyunsaturated fatty acids in response to Fe deficiency was observed in studies on *C. vulgaris* [40]. It is important to mention that under Fe or Mn-depleted conditions, in several microalgae species including *N. oceanica*, production polyunsaturated fatty acids can be qualitatively and quantitatively improved, which is really important since those specific fatty acids play an essential role in infant growth, cardiovascular health, as well as in cancer and inflammation [37]. Reduction of saturated fatty acids and polyunsaturated fatty acids, under P deprivation for *N. oceanica*, is confirmed also by an analogous study for *Picochlorum* sp. by El-Kassas [36]. The above results reveal the variability of phenotypes that microalgae species demonstrate regarding the fatty acid production with respect to macro- and micro-nutrients presence.

### 2.3. Effect of Culture Media Composition on Magnetic Harvesting Efficiency

**Figure 2:**
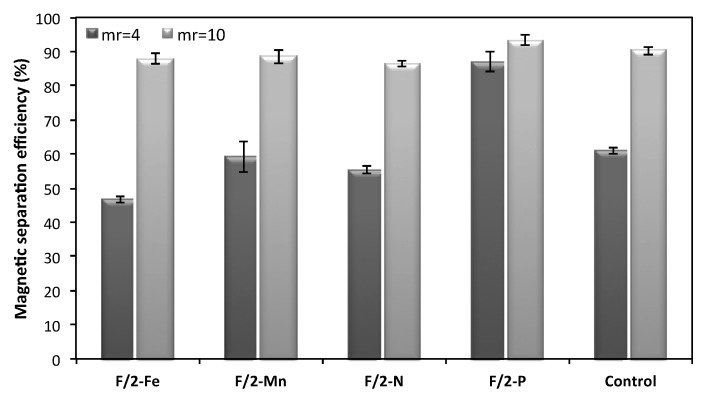
Influence of the culture medium composition on the separation efficiency of microalgae *Nannochloropsis oceanica* CCMP1779 at pH = 8 and m_p_/m_Nc_ equal to 4 (■) and 10 (■). The separation experiments were carried out in F/2 medium (control) and medium lacking each of the following components: NaH_2_PO_4_ (F/2-P), NaNO_3_ (F/2-N), FeCl_3_ (F/2-Fe), MnCl_2_ (F/2-Mn). The magnetic separation efficiency was estimated as a percentage of each one towards the respective component in complete culture medium in the two different mass ratios.

Beside *N. oceanica* growth, lipid biosynthesis and chlorophyll content, the culture medium ionicity affects the non-covalent interactions between magnetic microparticles and the cell surface, which ultimately are related to the separation efficiency of microalgae. The obtained results confirmed that the presence of specific ion types in the culture medium contributes significantly to the adhesion between microalgae and magnetic particles (Figure 2). Among all the nutrients considered in this study, P primarily affects the cell-magnetic particle interactions, since upon its depletion, harvesting efficiency was increased for both low and high mass ratios (m_p_/m_Nc_ = 4 and m_p_/m_Nc_ = 10), in contrast to the other nutrients where lower recovery efficiencies were obtained. Its effect is more intense when the magnetic particles-to-biomass mass ratio is four (m_p_/m_Nc_ = 4), where 87% of the microalgae cells are recovered compared to the control cells grown in complete medium (61%) and to other nutrients restriction (47%, 59% and 55% for Fe, Mn and N, respectively). Such a high harvesting efficiency is comparable to that obtained for the high mass ratio m_p_/m_Nc_ = 10, making viable and competitive the use of lower mass ratios as well. Specifically, for the case of m_p_/m_Nc_ = 10, the highest harvesting efficiency was also achieved in the absence of P (93%) and the lowest in the absence of N (87%), showing that the effect of the culture media composition was not so pronounced in this case. These findings are in line with Prochazkova et al. [14], who reported that the lack of phosphate ions in magnetic harvesting of *Chlorella vulgaris* led to the achievement of higher recovery efficiencies of microalgae. According to Prochazkova et al. [14], phosphorus anions can affect the surface charge of magnetic particles and can also block the functional binding sites of them by electrostatic attraction resulting in a negative effect on the recovery efficiencies. Hence, changes in electrostatic and van der Waals interactions caused by the presence or absence of ions can influence the separation of microalgae from the culture medium. However, taking into account that the separation mechanism that takes place in magnetic harvesting of microalgae is more complex and does not only involve electrostatic and/or van der Waals interactions, a general conclusion on the way ions affect the magnetic harvesting of *N. oceanica* cannot be drawn and needs to be further investigated [41]. Nevertheless, because of its simplicity the magnetic harvesting method could be easily implemented and scaled up to allow microalgae harvesting in large production facilities. Towards this direction, one important factor, amongst others, that needs further exploration could be a system for continuously collecting the flocculate formed by microalgae and magnetic microparticles. Indeed, a magnetic separator with high volume capacity working continuously seems to be essential when a large amount of biomass must be de-watered.

## 3. Materials and Methods

### 3.1. Organism and Growth Conditions

*Nannochloropsis oceanica* CCMP1779, purchased from the Provasoli-Guillard National Center for Culture of Marine Phytoplankton, was cultured in F/2 medium nutrients [42], composed of 0.075 g NaNO_3_; 0.005 g NaH_2_PO_4_·H_2_O; 3.15 × 10^−3^ g FeCl_3_·6H_2_O; 2.20 × 10^−5^ g ZnSO_4_·7H_2_O; 9.80 × 10^−6^ g CuSO_4_·5H_2_O; 4.36 × 10^−3^ g Na_2_EDTA·2H_2_O; 1.80.10^−4^ g MnCl_2_·4H_2_O; 1.00 × 10^−5^ g CoCl_2_·6H_2_O; 6.30 × 10^−6^ g Na_2_MoO_4_·2H_2_O; 5.00 × 10^−7^ g vitamin B_12_; 5.00 × 10^−7^ g biotin; 10^−4^ g thiamine·HCl (per liter). Carbon sources have been demonstrated to enhance biomass and lipid accumulation in both freshwater and marine microalgae [43] thus 0.5 g NaHCO_3_ L^−1^ was added at the beginning of the exponential phase.

Algae cells were harvested from a preculture, in the exponential phase, at a light intensity of 100 μmol photon/m^2^/s and were inoculated into 250 mL Erlenmeyer flask containing 100 mL sterilized seawater enriched with medium nutrients, under aseptic conditions. The cells grew on an orbital shaking incubator at 130 rpm and continual florescence light of 100 μmol photon/m^2^/s at the flask surface. Temperature and pH were adjusted at 20 °C and pH 8.0, respectively.

The experiments were carried out in F/2 medium and medium excluding one by one the following components: NaNO_3_ (F/2–N), NaH_2_PO_4_ (F/2–P), FeCl_3_ (F/2–Fe) and MnCl_2_ (F/2–Mn). All experiments were conducted in duplicate while samples were analyzed in triplicate.

### 3.2. Microalgae Dry Weight

Microalgae dry weight per liter (g L^−1^) was measured according to the method reported by Savvidou et al. [20]. Microalgae cells were collected by centrifugation of wet biomass at 7160× *g* (Beckman Model J2-21 Centrifuge) for 10 min. Prior to gravimetric analysis, in order to remove the salts, the centrifuged cells were washed twice with distilled water followed by drying at 105 °C overnight and the results were reported as g of dry cell weight (DCW) [44]. Biomass concentration values (X, g L^−1^) were used for determining the maximum biomass concentration (X_max_) on the 14th day of cultivation.

### 3.3. Extraction and Quantification of Chlorophyll

Chlorophyll a concentration can reflect the growth condition of microalgae. The pigment was extracted with methanol and determined by measuring the optical density at 665 nm using empirical correlations as described by Henriques et al. (2007) [43].

### 3.4. Determination of Lipid Content and Lipid Composition

*Nannochloropsis oceanica* oil extraction was carried out in two steps, subsequently lipid extraction and transesterification were performed. Lipid extraction was performed by adding 5 mL of the Folch solvent (2:1 of CHCl_3_: MeOH, v/v) to the dried biomass and homogenized in a sonication bath (VC 600, Sonics and Materials) at 25 °C. The procedure was repeated five times for 60 s each, through the homogenization process and after that, the sample was filtered and collected in a flask. KCl aqueous solution (0.88%) was added to the extracted solution in order to form two separate layers. The bottom layer (chloroform-rich phase) containing the lipid fraction was collected and then evaporated in a pre-weighed vial and dried under at 60 °C in a vacuum oven MODEL E28 (BINDER) to a constant weight. The solvents (absolute) were purchased from Sigma-Aldrich.

Transesterification was carried out in order to determine the fatty acid profile analysis of *Nannochloropsis oceanica*. The dried lipid was dissolved in toluene and 8% v/v hydrochloric acid in methanol was added into the solution. The solution was heated at 60 °C for 15 min, as described [45]. After heating, 2% w/v of a calcium chloride solution was added and the fatty acid methyl esters were extracted with 1 mL of n-hexane. The fatty acid profile was measured by using a 450 GC Varian gas chromatography coupled to a 220 IT MS Varian, mass spectrometer equipped with an Agilent J&W VF-23 ms capillary column (60 m × 0.25 mm, 0.25 μm film thickness) and with helium gas as the carrier at a constant flow of 1.4 mL/min. The GC-MS was operated at temperatures from 125 °C to 145 °C at a rate of 8 °C/min, kept constant for 26 min and then increased to 220 °C with a rate of 2 °C/min and kept constant for 9 min. The injection temperature was 250 °C. The lipid profiles of *N. oceanica* under different formulations of F/2 medium, were compared. Fatty acids were identified by comparison of retention times to these of a calibration standard solution (37 component FAME mix SUPELCO) and the mass spectra to the reference ones [20]. The data refer to triplicate analysis of at least two samples.

### 3.5. Kinetic and Yield Parameters

The specific growth rate (μ, d^−1^) of *N. oceanica* was calculated by Equation (1).

(1) $$\mu \;=\frac{1}{t}\cdot \ln\left(\frac{X_{m}}{X_{0}}\right)$$

where X_m_ and X_0_ are the concentrations of biomass at the end and at the beginning of a batch run, respectively, and t is the duration of the run.

The lipid content (Y, g lipids/100 g of DCW) of *N. oceanica* was estimated by Equation (2).

(2) $$Y\left(\%\right)=\frac{W_{L}}{W_{\mathrm{DCW}}}\times 100$$

where W_L_ and W_DCW_ are the weights of the extracted lipids and dry biomass, respectively.

The lipid productivity (P_L_, mg lipids/L/day) of *N. oceanica* was calculated by Equation (3).

(3) $$P_{L}=\frac{C_{L}}{t}$$

where C_L_ is the concentration of lipids at the end of the batch run and t is the duration of the run [30].

### 3.6. Magnetic Separation of Nannochloropsis Oceanica Microalgal Biomass

Recently, it was found that certain ions can affect the surface charge of magnetic beads and thus the recovery efficiency of microalgae. For this reason, the effect of the medium composition in the magnetic harvesting of microalgae *N. oceanica* was studied. Harvesting of microalgae grown in F/2 medium lacking components such as iron (FeCl_3_·4H_2_O), nitrogen (NaNO_3_), phosphorus (Na_2_H_2_PO_4_) and manganese (MnCl_2_·H_2_O) was performed. All harvesting experiments were conducted at the pH of the culture medium (pH 8–9). For comparison purposes, two different magnetic particles-to-biomass mass ratios (m_p_/m_Nc_), 4 and 10, were selected for the investigation of culture media effect on the recovery efficiency of microalgae. The selection of these two ratios was based on the findings of our previous work [19] where at m_p_/m_Nc_ = 4 and 10 the lowest and the highest separation efficiencies were obtained, respectively.

For the magnetic harvesting of *Nannochloropsis oceanica*, naked iron oxide (Fe_3_O_4_) magnetic microparticles were used. They were synthesized as proposed by Prochazkova et al. [14]. More details about their production and characterization can be found in a previous publication [19]. All magnetic harvesting experiments took place at environmental conditions. More specifically, a suspension of the magnetic particles in deionized water was added to that of microalgae and mixed by means of a mechanical stirrer for 10 min at 250 rpm. Then, the algal-particle flocs were harvested from the suspension medium by applying an external magnetic field via a permanent NdFeB magnet (50.8 mm × 50.8 mm × 25.4 mm) with a magnetic induction intensity of 12,600–12,900 G (N40, Supermagnete, Germany). Finally, the absorbance of the supernatant was measured at wavelength 750 nm by a spectrophotometer and the harvesting efficiency (%) was calculated as follows Equation (4):

(4) $$\mathrm{Harvesting}\;\mathrm{efficiency}\;(\%)=\frac{{\mathrm{OD}}_{0}-{\mathrm{OD}}_{1}}{{\mathrm{OD}}_{0}}\;\times \;100$$

where OD_0_ is the initial absorbance of the microalgal suspension before separation and OD_1_ the absorbance of the supernatant after the magnetic cell separation.

### 3.7. Statistical Analysis

In statistical analysis, the Student’s *t* test compares the actual difference between means in relation to the variation in the data. The results were considered significant if the *p*-value was lower than 0.05.

## 4. Conclusions

The depletion effect of single macro- and micro-nutrients like Fe^+3^, Mn^+2^, P and N on growth, fatty acid composition and magnetic harvesting of the microalgae cells *N. oceanica* CCMP1779 was investigated. Enhanced biomass production was achieved by P and Fe depletion compared to Mn and N restriction. Phosphorus depletion resulted in lower lipid content (23.7%) while upon N deprivation higher lipid content (41.5%) was achieved, a finding that is in agreement with most studies performed in various microalgae species. Phosphorus restriction favors monounsaturated fatty acid production (40.24%), while N depletion increases the amount of saturated fatty acids (60.6%). Higher polyunsaturated fatty acids were achieved by Fe or Mn depletion. The magnetic harvesting efficiency at m_p_/m_Nc_ = 4 of *N. oceanica* cells grown in P deprivation was significantly higher (87%) compared to the other nutrients starvation or even to complete medium and comparable to the one obtained with m_p_/m_Nc_ = 10 (93%). These encouraging findings on magnetic separation technology, especially in low m_p_/m_Nc_ values, offer a great potential for an efficient, time and energy saving method for algal harvesting without environmental pollution while the optimization of growth medium nutrients gives the advantage of the preferential fatty acid production of various applications. Taking into account all presented data it is evident that cell growth, separation efficiency and fatty acid production of *N. oceanica* depend upon culture medium composition. Herein a suitable tool is proposed for optimizing growth media of microalgae cells, shifting the metabolism to produce specific fatty acids depending on pharmaceutical or biodiesel industry demands.

## Figures and Tables

**Table 1:** Production parameters of *N. oceanica* CCMP1779 cultivation.

Medium Composition	F/2-P	F/2-N	F/2-Fe	F/2-Mn	Control
μ (d^−1^)	0.274	0.215	0.286	0.253	0.278
X_max_ (g L^−1^)	0.29	0.26	0.29	0.25	0.31
P_x_ (mg L^−1^ d^−1^)	18.55	16.44	18.15	15.37	20.01
Y (%_DCW_)	23.7	41.5	33.4	34.5	37.5
P_L_ (mg L^−1^ d^−1^)	4.0	6.4	5.7	5.0	6.8
Chl (μg mL^−1^)	0.95	1.41	4.85	2.76	2.70

F/2 medium (control) and medium lacking each of the following components: NaH_2_PO_4_ (F/2-P), NaNO_3_ (F/2-N), FeCl_3_ (F/2-Fe), MnCl_2_ (F/2-Mn). Specific growth rates-μ, max dry biomass-X_max_, biomass productivity-Ρ_X_, lipid content-Υ, lipid productivity-P_L_, chlorophyll content-Chl. Values are the means of three measurements and the standard deviation was below 5% in all cases.

**Table 2:** Fatty Acids profile of oil extracted from *Nannochloropsis oceanica* biomass cultured in different medium compositions.

Medium Composition	F/2-P	F/2-N	F/2-Fe	F/2-Mn	Control
**Total FAs content ^1^**	10.9 ± 0.5	7.3 ± 0.8	11.7 ± 0.4	11.7 ± 0.6	9.22 ± 0.25
	**% Ratio to total FA**				
**Saturated Fatty Acids (SFAs)**					
C12:0	1.74 ± 0.2	1.64 ± 0.1	2.15 ± 0.3	2.66 ± 0.2	2.39 ± 0.2
C14:0	7.15 ± 0.6	10.26 ± 0.8	8.24 ± 0.7	9.09 ± 0.5	8.46 ± 0.4
C15:0	1.37 ± 0.1	1.78 ± 0.3	1.97 ± 0.2	2.06 ± 0.3	1.84 ± 0.3
C16:0	36.21 ± 0.7	41.04 ± 0.8	35.19 ± 0.7	33.45 ± 0.9	37.64 ± 1.1
C18:0	5.68 ± 0.4	4.79 ± 0.2	4.46 ± 0.4	5.75 ± 0.5	3.90 ± 0.5
Sum SFAs	53.35 ± 1.2	60.60 ± 0.9	53.56 ± 0.8	54.72 ± 0.9	55.75 ± 1.1
**Monounsaturated Fatty Acids (MUFAs)**					
C16:1 *n*-7	26.31 ± 0.8	22.16 ± 0.5	23.86 ± 0.6	21.10 ± 0.7	23.43 ± 0.5
C18:1 *n*-9	13.93 ± 0.4	10.12 ± 0.5	7.81 ± 0.2	7.20 ± 0.5	10.95 ± 0.4
Sum MUFAs	40.24 ± 1.1	32.38 ± 0.8	31.67 ± 0.8	28.30 ± 1.6	34.38 ± 0.6
**Polyunsaturated Fatty Acids (PUFAs)**					
C18:2 *n*-6	1.74 ± 0.2	1.78 ± 0.3	2.40 ± 0.5	2.83 ± 0.4	2.17 ± 0.3
C20:4 *n*-3 (ETA)	1.65 ± 0.3	1.92 ± 0.3	3.52 ± 0.5	4.03 ± 0.1	2.49 ± 0.3
C20:5 *n*-3 (EPA)	3.02 ± 0.4	3.42 ± 0.2	8.84 ± 0.3	10.12 ± 0.7	5.21 ± 0.4
Sum PUFAs	6.42 ± 0.8	7.11 ± 0.5	14.76 ± 0.2	16.98 ± 0.6	9.87 ± 0.4

F/2 medium (control) and medium lacking each of the following components: NaH_2_PO_4_ (F/2-P), NaNO_3_ (F/2-N), FeCl_3_ (F/2-Fe), MnCl_2_ (F/2-Mn). ^1^ 1 g of fatty acids (FAs)/100 g of lipids. Results are expressed as the mean ± SD (n = 3).
